# Church and Social Welfare

**Published:** 1920-02-14

**Authors:** 


### Church and Social Welfare.

The report of the Archbishop of Canterbury's Committee on the ways in which clergy and Churchmen generally can best co-operate with the State in all matters concerning the social life of the community, points out how the whole system of our social life has been changed by the machinery adopted since 1914 and how it requires, in addition to salaried officials, a vast number of unpaid volunteers. The Committee urges that social study and social work are to be looked upon as natural parts of a clergyman's vocation, for it is his special duty to take the lead in the application of the Christian faith to social and industrial practices. A first-hand and practical knowledge of social legislation and of the general working of the social system will make the clergyman's indirect social action all the more effective and extend his influence for good over a wider circle. Theological colleges should give systematised teaching on social subjects to their students, and the study of these subjects should be continued after ordination.

The Committee holds that all the clergy should normally be regarded as "under training" for the three years following their ordination as deacons. It would be of inestimable value if the junior clergy, having had some practical experience of parish work, could undertake definite social study in connection with a University course.

During the first three years of his ministry every clergyman working in a town who has not yet already obtained a diploma should be required to attend an approved course in social science of at least six months, and, if practicable, in a University town or under University direction. A director of social studies should be appointed in each diocese, who should encourage the study of social questions among the clergy.

